# Comparative Evaluation of Citric Acid, Chlorhexidine, and Their Combination as Root Canal Irrigants on the Push‐Out Bond Strength of Resin and Calcium Silicate‐Based Sealers to Root Canal Dentin

**DOI:** 10.1155/ijod/7091912

**Published:** 2026-09-21

**Authors:** Laasya Shettigar, Tina Puthen Purayil, Kishore Ginjupalli, Nidambur Vasudev Ballal

**Affiliations:** ^1^ Department of Conservative Dentistry and Endodontics, Manipal College of Dental Sciences, Manipal Academy of Higher Education, Manipal, India, manipal.edu; ^2^ Department of Dental Materials, Manipal College of Dental Sciences, Manipal Academy of Higher Education, Manipal, India, manipal.edu

## Abstract

**Background:**

Root canal irrigants can alter root canal dentin and influence sealer adhesion. With increasing use of bioceramic sealers, understanding the effects of irrigants on bonding is essential for endodontic success. This in vitro study evaluated the effects of 1% chlorhexidine (CHX), 10% citric acid (CA), and their combination (CACHX) on the push‐out bond strength (POBS) of AH Plus and BioRoot RCS to root canal dentin.

**Methods:**

Eighty single‐rooted premolars with a single canal were selected and divided into eight groups (*n* = 10) based on the final irrigation protocol: CA, CHX, CACHX, or distilled water. Root slices (2 mm) were obtained from the middle third. Groups A1–A4 were obturated with AH Plus and B1–B4 with BioRoot RCS. POBS was assessed using a universal testing machine, and failure modes were analyzed under a stereomicroscope. Normality was confirmed using the Shapiro–Wilk test. Intergroup comparisons were performed using independent *t*‐tests, and intragroup comparisons were performed using one‐way analysis of variance (ANOVA) with Tukey’s post hoc test.

**Results:**

The final irrigant significantly affected bond strength (*p*  < 0.001). AH Plus showed higher POBS than BioRoot RCS with CACHX (*p* = 0.008). BioRoot RCS exhibited the highest POBS with CA and the lowest with CHX (*p*  < 0.05). AH Plus showed the maximum bond strength with CHX, with no reduction when combined with CA (*p*  > 0.05). Cohesive failures predominated in AH Plus, whereas BioRoot RCS showed mixed failures.

**Conclusion:**

In this in vitro study, root canal irrigants significantly influenced sealer bond strength. AH Plus exhibited the highest bond strength with CHX, whereas BioRoot RCS performed best with CA. The combination of CA and CHX did not adversely affect AH Plus and may be considered a suitable irrigant protocol for this sealer.

## 1. Introduction

The primary objective of root canal therapy is to obtain a fluid‐tight seal within the root canal to prevent bacterial reinfection effectively [1]. Thorough disinfection of the root canal is achieved through a combination of mechanical instrumentation of the canal, irrigation, and the use of intracanal medicaments [2]. Since mechanical instrumentation alone cannot completely clean the complex root canal anatomy, the selection of an ideal irrigant is particularly important in disinfecting areas that are inaccessible to mechanical instrumentation and improving treatment outcomes [3].

Sodium hypochlorite (NaOCl) is considered the gold standard irrigant because of its superior organic tissue‐dissolving properties [4]. Chlorhexidine (CHX), known for its antimicrobial efficacy, is used as an irrigant and an intracanal medicament [5–7]. However, neither NaOCl nor CHX is capable of removing the inorganic portion of the smear layer. Therefore, chelating agents such as ethylenediaminetetraacetic acid (EDTA), citric acid (CA), and maleic acid are recommended for smear layer removal [8]. CA is a weak tricarboxylic acid with chelating properties that facilitate smear layer removal while aiding in the disinfection of infected root dentin. Among the concentrations evaluated, 10% CA has been shown to effectively remove the smear layer with minimal dentinal erosion, making it a suitable chelating agent for root canal irrigation [9].

Although the sequential use of NaOCl and EDTA is widely accepted during root canal treatment, their chemical interaction reduces the available chlorine in NaOCl, diminishing its tissue‐dissolving ability and potentially affecting the bonding performance of resin‐based sealers [6, 10, 11]. Consequently, alternative irrigation strategies capable of providing both antimicrobial activity and smear layer removal have been explored.

Prado et al. [12] demonstrated that combining 2% CHX with 10% CA produced a chemically stable solution without precipitate formation. Subsequently, González‐López et al. [13] reported that CHX did not interfere with the demineralizing action of CA. More recently, Scelza et al. [14] demonstrated that the combination of 1% CHX and 10% CA exhibited effective antimicrobial activity, smear layer removal, and acceptable cytocompatibility, suggesting its potential use as a single irrigating solution. Although the biological properties of this irrigant combination have been investigated, its influence on the adhesion of contemporary root canal sealers to dentin remains unclear.

Root canal sealers play a vital role in filling spaces between the gutta‐percha and the root canal as well as the lateral and accessory canals [1]. AH Plus is regarded as the gold standard epoxy resin‐based sealer because of its excellent sealing ability and favorable physicochemical properties. BioRoot RCS, a calcium silicate‐based sealer, offers biocompatibility, antibacterial properties, and the ability to set inside a wet canal [15]. It has also been reported to exhibit push‐out bond strength (POBS) to root canal dentin comparable to that of the AH Plus sealer [16].

Since the interaction between irrigants and sealers may influence the long‐term integrity of root canal obturation, evaluating the effect of 10% CA–1% CHX on sealer adhesion is clinically relevant. Therefore, this study aimed to evaluate and compare the effects of irrigation with 1% CHX, 10% CA, and their combination on the POBS of AH Plus and BioRoot RCS sealers to root canal dentin. The null hypothesis was that the combination of CA and CHX as a root canal irrigant would not affect the POBS of either sealer to root canal dentin.

## 2. Materials and Methods

The study was conducted after obtaining approval from the Institutional Ethics Committee (IEC2: 218/2023) and in accordance with the principles of the Declaration of Helsinki.

### 2.1. Sample Size Calculation

The required sample size was calculated using 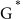
Power 3.1.9.7 to support application of one‐way analysis of variance (ANOVA) for comparison of mean POBS among the four final irrigant protocols within each sealer group. Based on an assumed effect size of Cohen’s *f* = 0.6, a significance level of *α* = 0.05, and power (1‐*β*) = 0.80, a minimum of nine specimens per group (total *n* = 36) was required for each sealer to detect meaningful differences. The present study included a total of 80 specimens, distributed equally across 8 sealer–irrigant groups (*n* = 10 per group), thereby exceeding the minimum required sample and ensuring adequate statistical power for both sealers.

### 2.2. Specimen Preparation

Eighty freshly extracted, noncarious, single‐rooted human premolars with a single straight canal and fully formed apices, extracted for orthodontic reasons, were obtained from the Department of Oral and Maxillofacial Surgery, Manipal College of Dental Sciences, Manipal. Teeth with cracks, root resorption, previous endodontic treatment, calcified canals, or multiple canals were excluded from the study. Following extraction, the teeth were cleaned of soft tissue remnants and stored in a thymol solution (KMC Pharmacy, Manipal, Karnataka, India) until use. The crown portion of each tooth was decoronated using a diamond disc (Horico Dental Hopf, Ringleb & Co. GmbH & Cie, Berlin, Germany) to obtain 14‐mm‐long root segments. To standardize specimens and minimize anatomical variability, each tooth contributed only one 2‐mm dentin slice. Each tooth was used as one independent sample for statistical analysis. The pulp tissue was removed using a broach (MANI Inc., Tochigi, Japan). The working length was established by inserting a No. 15 K‐file (MANI Inc., Tochigi, Japan) to the root canal terminus and subtracting 1 mm from this measurement. The root canal orifice was enlarged using GG drills (MANI Inc., Tochigi, Japan) to size No. 3. The root canals were then instrumented using ProTaper nickel–titanium rotary instruments (Dentsply Sirona, Tulsa, OK, USA), and each canal was enlarged to F3 size at the working length. The samples were irrigated with 5 mL of 2.5% NaOCl (KMC Pharmacy, Manipal, Karnataka, India) between each file change.

### 2.3. Irrigation Protocol

Following instrumentation, each specimen was assigned a number and randomly allocated into eight experimental groups (*n* = 10) using the lottery method according to the final irrigation protocol (Table 1).

**Table 1:** Division of groups based on the irrigation protocol.

Groups A: AH Plus	Group B: BioRoot RCS
Group A1: 10% CA (5 min)	Group B1: 10% CA (5 min)
Group A2: 1% CHX (5 min)	Group B2: 1% CHX (5 min)
Group A3: CACHX (5 min)	Group B3: CACHX (5 min)
Group A4: DW (5 min)	Group B4: DW (5 min)

A total of 10% CA was prepared by dissolving 5 g of CA (KMC Pharmacy, Manipal, Karnataka, India) in DW and adjusting the final volume to 50 mL. Similarly, 0.5 g (500 mg) of CHX powder was dissolved in DW, and the final volume was adjusted to 50 mL to obtain a 1% (w/v) CHX solution (500 mg/50 mL = 1 g/100 mL = 1% w/v). Both solutions were homogenized by continuous stirring until complete dissolution was achieved. Equal volumes of 10% CA and 1% CHX were then mixed and homogenized until a clear, transparent solution was obtained. No visible precipitate was noted during the mixing of 1% CHX and 10% CA.

The irrigation was performed using 5 mL of the respective irrigant with a 5‐mL syringe and a 30‐gauge side‐vented irrigation needle. The irrigant was delivered at a standardized flow rate of 0.25 mL/s, with the needle positioned 1 mm short of the working length. Following irrigation, all specimens were rinsed with 5 mL of distilled water for 1 min to remove residual remnants from the canal system.

After irrigation, the root canals were dried with paper points (Dentsply Sirona, Tulsa, OK, USA). Each root was embedded in cold‐cure acrylic resin using a custom‐made mold (15 mm in diameter). After polymerization, the roots were sectioned at the middle third using a diamond disc (Horico Dental Hopf, Ringleb & Co. GmbH & Cie, Berlin, Germany) to obtain 2‐mm‐thick dentin slices. Slice thickness was measured using a digital Vernier caliper (ABSOLUTE Digimatic Caliper Series 500; Mitutoyo Corp., Kanagawa, Japan). The canals in groups A1, A2, A3, and A4 were filled with AH Plus sealer (Dentsply Sirona, De‐Trey‐Str. Konstanz, Germany), whereas those in groups B1, B2, B3, and B4 were filled with BioRoot RCS (Septodont, Saint‐Maur‐des‐Fossés, France). Both sealers were mixed according to the manufacturer’s guidelines before placement into the canal spaces. The samples were then incubated at 37°C under 100% humidity for 24 h to allow the complete setting of the sealers.

### 2.4. POBS Analysis

The push‐out test was performed with a universal testing machine (Bluehill Universal Software, Instron Corp., Norwood, MA, USA). Each sample was positioned on the testing platform, and a stainless‐steel plunger measuring 0.9 mm in diameter was carefully aligned to contact only the sealer without touching the surrounding dentin. A compressive load was applied in the apicocoronal direction at a crosshead speed of 1 mm/min until bond failure occurred. The maximum load at failure was recorded in Newtons (N).

The POBS in MPa was measured using the following formula:

POBS MPa= Force at which bond failure occursN/adhesion surface areamm2.

The adhesion surface area was calculated by the following formula:

Adhesion surface areamm2=2×π×r×h.

where *r* = (r1 + r2) /2, *r* is the root canal radius, *r*_1_ is the root canal radius at the coronal side, *r*_2_ is the root canal radius at the apical side, *π* is the constant 3.14, and *h* is the thickness of the dentin slice.

### 2.5. Failure Mode Analysis

Following POBS analysis, the specimens were examined under a stereomicroscope (Leica Microsystems, Wetzlar, Germany) at ×40 magnification to determine the mode of failure. Failure modes were recorded as adhesive, cohesive, or mixed based on the predominant pattern observed.

• Adhesive failure: failure occurring predominantly at the sealer–dentin interface.
• Cohesive failure: failure occurring predominantly within the sealer.
• Mixed failure: a combination of adhesive and cohesive failures.

The POBS test and failure mode assessment were performed by independent investigators who were blinded to the group allocation.

### 2.6. Statistical Analysis

Statistical analysis was performed using SPSS software (Version 23, IBM Corp., Armonk, NY, USA). The normality of the data distribution was assessed using the Shapiro–Wilk test, and the homogeneity of variances was verified using Levene’s test. A two‐way ANOVA was used to evaluate the effects of sealer type and irrigation protocol on POBS, as well as their interaction. Because a significant interaction between the two factors was observed, simple effects were analyzed separately for each sealer using one‐way ANOVA. Post hoc pairwise comparisons were performed using Tukey’s honestly significant difference (HSD) test. The level of statistical significance was set at *p*  < 0.05.

## 3. Results

All 80 specimens were included in the analysis. The Shapiro–Wilk test confirmed that the POBS data were normally distributed, and Levene’s test showed the homogeneity of variances. The mean POBS values for each sealer under the different irrigation protocols are presented in Table 2.

**Table 2:** Mean push‐out bond strength (MPa) for each sealer and irrigant protocol.

Sealer	BioRoot RCS		AH Plus	
Irrigant	Mean (MPa)	SD	Mean (MPa)	SD
Distilled water	11.68	1.21	14.02	2.75
CHX	4.89	0.72	16.76	1.72
Citric acid	14.01	1.62	12.72	0.95
CACHX	12.53	1.23	14.53	1.71

BioRoot RCS demonstrated the highest bond strength after CA irrigation (14.01 MPa), followed by CACHX and distilled water, whereas CHX produced a marked reduction in adhesion (4.89 MPa). AH Plus showed the highest mean bond strength following CHX irrigation (16.76 MPa), whereas CA showed the lowest value for this sealer (12.72 MPa).

### 3.1. Effect of Sealer Type and Irrigation Protocol on POBS

Two‐way ANOVA demonstrated that the sealer type and irrigation protocol had significant effects on POBS. A significant interaction between sealer type and irrigation protocol was also observed, indicating that the effect of the irrigation protocol differed according to the sealer used (all *p*  < 0.001) (Table 3). Sealer type accounted for 60.2% of the variability in POBS (partial *η* ^2^ = 0.602), whereas the irrigation protocol accounted for 33.5% of the variability (partial *η* ^2^ = 0.335). The interaction between sealer type and irrigation protocol showed a partial *η* ^2^ of 0.724.

**Table 3:** Two‐way ANOVA analysis of the effects of irrigant, sealer, and their interaction on push‐out bond strength.

Source	Mean square	*F*	Sig.	Partial eta squared
Irrigant	30.998	12.117	<0.001	0.335
Sealer	278.280	108.782	<0.001	0.602
Irrigant × sealer	160.680	62.811	<0.001	0.724

### 3.2. Comparison of Irrigation Protocols Within Each Sealer Group

Within the BioRoot RCS group, CA exhibited significantly higher POBS than distilled water (*p* = 0.001) and CHX (*p*  < 0.001). The difference between CA and CACHX was not statistically significant (*p* = 0.054). CHX showed significantly lower POBS than the other irrigation protocols (*p*  < 0.001). Distilled water and CACHX did not differ significantly (*p* = 0.423).

Within the AH Plus group, CHX showed significantly higher POBS than distilled water (*p* = 0.013) and CA (*p*  < 0.001). The difference between CHX and CACHX was not statistically significant (*p* = 0.057). CA showed significantly lower POBS than CHX, but it did not differ significantly from distilled water (*p* = 0.434) or CACHX (*p* = 0.163). Distilled water and CACHX did not differ significantly (*p* = 0.930) (Table 4).

**Table 4:** Tukey HSD multiple comparisons of push‐out bond strength among irrigants for each sealer.

Sealer	Comparison	Mean difference (I–J)	Std. error	*p*‐Value	95% CI
BioRoot RCS	DW vs. CHX	6.79136	0.55290	<0.001	[5.3023, 8.2804]
BioRoot RCS	DW vs. CA	−2.32403	0.55290	0.001	[−3.8131, −0.8350]
BioRoot RCS	DW vs. CACHX	−0.85310	0.55290	0.423	[−2.3422, 0.6360]
BioRoot RCS	CHX vs. CA	−9.11539	0.55290	<0.001	[−10.6045, −7.6263]
BioRoot RCS	CHX vs. CACHX	−7.64446	0.55290	<0.001	[−9.1336, −6.1553]
BioRoot RCS	CA vs. CACHX	1.47093	0.55290	0.054	[−0.0182, 2.9601]
AH Plus	DW vs. CHX	−2.74885	0.84709	0.013	[−5.0303, −0.4674]
AH Plus	DW vs. CA	1.29168	0.84709	0.434	[−0.9897, 3.5731]
AH Plus	DW vs. CACHX	−0.51315	0.84709	0.930	[−2.7946, 1.7683]
AH Plus	CHX vs. CA	4.04053	0.84709	<0.001	[1.7591, 6.3220]
AH Plus	CHX vs. CACHX	2.23570	0.84709	0.057	[−0.0457, 4.5171]
AH Plus	CA vs. CACHX	−1.80483	0.84709	0.163	[−4.0862, 0.4765]

### 3.3. Comparison of Sealers Within Each Irrigation Protocol

When the two sealers were compared within each irrigation protocol, AH Plus showed higher mean POBS than BioRoot RCS after distilled water, CHX, and CACHX irrigation. In contrast, BioRoot RCS showed a higher mean POBS than AH Plus after CA irrigation.

### 3.4. Analysis of Failure Modes

**Figure 1:**
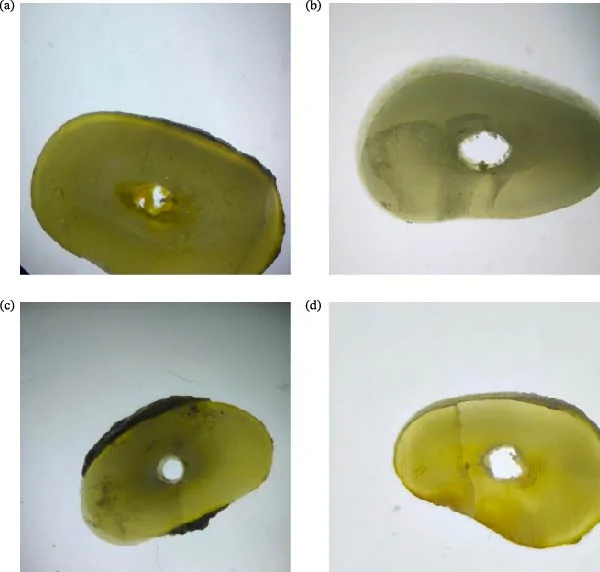
Representative stereomicroscopic images showing failure modes after push‐out bond strength testing. (a) Cohesive failure in AH Plus sealer. (b) Mixed failure in BioRoot RCS. (c) Adhesive failure in BioRoot RCS. (d) Cohesive failure in BioRoot RCS.

The AH Plus sealer showed predominantly cohesive failures across all groups, whereas BioRoot RCS showed a combination of cohesive, adhesive, and mixed failures (Table 5; Figure 1). Failure mode distributions were compared using the Fisher–Freeman–Halton exact test. No significant differences were observed between AH Plus and BioRoot RCS within the DW (*p* = 0.087), CHX (*p* = 0.173), CA (*p* = 0.076), or CACHX (*p* = 0.150) groups (Table 6).

**Table 5:** Failure mode distribution after the push‐out bond strength test (*n* = 10).

Sealer	Irrigant	Failures					
		Cohesive		Adhesive		Mixed	
		Percentage (%)	Frequency	Percentage (%)	Frequency	Percentage (%)	Frequency
AH Plus	DW	100	10	0	0	0	0
	CHX	90	9	10	1	0	0
	CA	90	9	0	0	10	1
	CACHX	80	8	10	1	10	1
BioRoot RCS	DW	60	6	20	2	20	2
	CHX	50	5	30	3	20	2
	CA	40	4	30	3	30	3
	CACHX	30	3	40	4	30	3

**Table 6:** Fisher–Freeman–Halton exact test comparing failure mode distribution between AH Plus sealer and BioRoot RCS for each irrigant.

Irrigant	*p*‐Value	Significance
DW	0.087	Not significant
CHX	0.173	Not significant
CA	0.076	Not significant
CACHX	0.150	Not significant

## 4. Discussion

The present study showed that 10% CA produced the highest POBS for BioRoot RCS, whereas 1% CHX produced the lowest POBS. In contrast, the AH Plus sealer showed the highest POBS with 1% CHX. Since the POBS values of the CACHX group did not differ significantly from those of the distilled water and CA groups for either sealer, the null hypothesis that the combination of CA and CHX would not affect the POBS of either sealer was accepted. For BioRoot RCS, however, the CACHX group produced significantly higher POBS than CHX alone, indicating that CA may lessen the marked reduction in bond strength otherwise caused by CHX for this sealer.

The POBS test used to assess bond strength closely replicates the clinical situation as the tested materials are placed inside the canals and the load is applied in a direction perpendicular to the dentinal tubules [17, 18]. Isolated 2‐mm dentin slices were selected to reduce anatomical variability and provide a more controlled and reproducible model for assessing bond strength. This approach helps isolate the effect of the tested materials on dentin while minimizing confounding factors related to differences in root canal morphology. However, this model has important limitations. It does not reproduce the full three‐dimensional complexity of the root canal system, the presence of complete obturation, or the clinical stresses present in vivo. Therefore, POBS should be interpreted as a comparative in vitro measure of adhesion rather than a direct predictor of clinical performance. In addition, thermoplastic gutta‐percha was not included because it may deform during testing, which could affect measurement accuracy; therefore, the canals were filled with sealer alone to permit a more reliable assessment of bond strength [19–21].

AH Plus, an epoxy resin‐based sealer, bonds to dentin through chemical interaction with dentinal collagen, which may contribute to its higher POBS [22]. The results of the study indicate that the AH Plus sealer showed higher POBS values than the BioRoot RCS sealer across all the irrigant groups, except the CA group. This finding is in agreement with previous reports showing higher POBS for AH Plus than for calcium silicate‐based sealers after irrigation with CHX‐containing protocols [23].

CHX is a widely used antimicrobial agent and a known matrix metalloproteinase (MMP) inhibitor. By inhibiting MMP activity, CHX may help preserve dentin collagen and improve bond stability, which may explain the higher POBS observed for AH Plus with 1% CHX in this study [24]. BioRoot RCS showed the lowest POBS with 1% CHX, which is consistent with the findings of Ghatole et al. [25], who reported reduced POBS when BioRoot RCS was used with CHX. BioRoot RCS forms a mineral infiltration zone that promotes adhesion by forming cement tags [26, 27]. However, Srivastava et al. [16] reported contrasting results and concluded that BioRoot RCS had a greater POBS than AH Plus sealer and that irrigation with 2% CHX resulted in a greater POBS of BioRoot RCS. They suggested that this could be because of the ability of CHX to improve surface energy and wettability, thereby improving adhesion for the hydrophilic BioRoot RCS [16].

In the present study, irrigation with CA showed the highest POBS for the BioRoot RCS group. CA has been shown to remove the smear layer effectively, and smear‐layer removal may facilitate sealer penetration into dentinal tubules and improve interfacial adaptation [28–30]. BioRoot RCS is a tricalcium silicate‐based bioceramic sealer, and its bioactive and hydrophilic characteristics may contribute to its bonding behavior [31]. In contrast, the slightly lower POBS observed for AH Plus with CA may reflect differences in sealer composition and dentin interaction between epoxy resin and bioceramic sealers [32, 33].

The combination of NaOCl and EDTA is known to cause dentinal erosion and collagen degradation [34]. González‐López et al. [13] showed that 1% CHX did not reduce the decalcifying ability of 10% CA, indicating that the combination could be an irrigation option. Dewi et al. [35] reported that CA‐modified CHX showed smear‐layer removal efficacy comparable to 17% EDTA and also demonstrated antimicrobial and antibiofilm activity [36]. In the present study, the CA‐CHX group did not differ significantly in POBS from CA alone or distilled water for either sealer. However, for BioRoot RCS, the CA‐CHX combination produced significantly higher POBS than CHX alone, suggesting that CA may reduce the adverse effect of CHX on BioRoot RCS bonding. For AH Plus, the CA‐CHX combination did not differ significantly from CHX alone, indicating that adding CA did not compromise the elevated bond strength associated with CHX for this sealer. This may be attributable to the combined effects of smear‐layer removal by CA and the antimicrobial properties of CHX. Although a higher POBS indicates greater resistance to dislodgement under laboratory conditions, no clinically accepted threshold has been established for an ideal POBS value; therefore, POBS should be interpreted as a comparative in vitro parameter rather than a direct predictor of clinical success.

In the present study, the AH Plus sealer exhibited cohesive failures across all irrigant groups, whereas BioRoot RCS showed a combination of adhesive, cohesive, and mixed failures. This finding is broadly consistent with earlier studies reporting stronger dislodgement resistance for AH Plus and more variable failure patterns for bioceramic sealers [23, 37]. The cohesive failures seen with AH Plus may reflect stronger dentin‐sealer interaction, which has been associated with chemical bonding to dentinal collagen [38].

The primary limitation of this study is its experimental setup. In a controlled environment, calcium silicate‐based sealers may take longer to set due to the 100% humidity in an incubator, whereas in a natural setting, dentinal tubules may contain fluid that could influence the setting process. In addition, the core material was not incorporated during POBS testing because gutta‐percha is thermoplastic and may deform under load. Therefore, further studies are warranted to confirm the results under more clinically relevant conditions.

## 5. Conclusion

Within the limitations of this in vitro study, the final irrigation protocol influenced the POBS of both sealers. AH Plus showed the highest POBS with 1% CHX, whereas BioRoot RCS showed the highest POBS with 10% CA and the lowest with 1% CHX. For BioRoot RCS, combining CA with CHX significantly improved POBS relative to CHX alone, suggesting that this combination may help reduce CHX’s adverse effect on this sealer; for AH Plus, the combination did not compromise the elevated bond strength associated with CHX alone. Failure mode analysis showed predominantly cohesive failures for AH Plus, whereas BioRoot RCS exhibited cohesive, adhesive, and mixed failures. These findings may not fully translate to clinical outcomes, as the experimental conditions do not completely replicate the oral environment.

## Author Contributions

**Laasya Shettigar:** conceptualization, methodology, investigation, data curation, formal analysis, writing – original draft, writing – review and editing. **Tina Puthen Purayil**: conceptualization, data curation, validation, writing – review and editing. **Kishore Ginjupalli**: validation, writing – review and editing. **Nidambur Vasudev Ballal**: writing – review and editing.

## Funding

This study did not receive any funding.

## Disclosure

All authors have read and approved the final version of the manuscript. The corresponding author had full access to all the data in this study and takes complete responsibility for the integrity of the data and the accuracy of the data analysis.

## Conflicts of Interest

The authors declare no conflicts of interest.

## Data Availability

The data supporting the findings of this study are available from the corresponding author upon reasonable request.

## References

[1] Park M.-G. , Kim I.-R. , Kim H. J. , Kwak S. W. , and Kim H.-C. , Physicochemical Properties and Cytocompatibility of Newly Developed Calcium Silicate-Based Sealers, Australian Endodontic Journal. (2021) 47, no. 3, 512–519, 10.1111/aej.12515. 33894082

[2] Ballal N. V. , Gandhi P. , Shenoy P. A. , and Dummer P. M. H. , Evaluation of Various Irrigation Activation Systems to Eliminate Bacteria From the Root Canal System: A Randomized Controlled Single-Blinded Trial, Journal of Dentistry. (2020) 99, 10.1016/j.jdent.2020.103412, 103412. 32585261

[3] Haapasalo M. , Shen Y. , Wang Z. , and Gao Y. , Irrigation in Endodontics, British Dental Journal. (2014) 216, no. 6, 299–303, 10.1038/sj.bdj.2014.204. 24651335

[4] Jaiswal N. , Sinha D. J. , Singh U. P. , Singh K. , Jandial U. A. , and Goel S. , Evaluation of Antibacterial Efficacy of Chitosan, Chlorhexidine, Propolis and Sodium Hypochlorite on *Enterococcus faecalis* Biofilm: An In Vitro Study, Journal of Clinical and Experimental Dentistry. (2017) 9, no. 9, 10.4317/jced.53777, e1066. PMC5650207 29075407

[5] Russell A. D. and Day M. J. , Antibacterial Activity of Chlorhexidine, Journal of Hospital Infection. (1993) 25, no. 4, 229–238, 10.1016/0195-6701(93)90109-D. 7907620

[6] Calişkan M. K. , Türkün M. , and Alper S. , Allergy to Sodium Hypochlorite During Root Canal Therapy: A Case Report, International Endodontic Journal. (1994) 27, no. 3, 163–167, 10.1111/j.1365-2591.1994.tb00247.x. 7995650

[7] Zehnder M. , Root Canal Irrigants, Journal of Endodontics. (2006) 32, no. 5, 389–398, 10.1016/j.joen.2005.09.014. 16631834

[8] Gómez-Delgado M. , Camps-Font O. , Luz L. , Sanz D. , and Mercadé M. , Update on Citric Acid use in Endodontic Treatment: A Systematic Review, Odontology. (2023) 111, no. 1, 1–19, 10.1007/s10266-022-00744-2. 36220913

[9] Ballal N. V. , Roy A. , and Zehnder M. , Effect of Sodium Hypochlorite Concentration in Continuous Chelation on Dislodgement Resistance of an Epoxy Resin and Hydraulic Calcium Silicate Sealer, Polymers. (2021) 13, no. 20, 10.3390/polym13203482, 3482. PMC8537795 34685241

[10] Grawehr M. , Sener B. , Waltimo T. , and Zehnder M. , Interactions of Ethylenediamine Tetraacetic Acid With Sodium Hypochlorite in Aqueous Solutions, International Endodontic Journal. (2003) 36, no. 6, 411–415, 10.1046/j.1365-2591.2003.00670.x. 12801288

[11] Paulson L. , Ballal N. V. , and Bhagat A. , Effect of Root Dentin Conditioning on the Pushout Bond Strength of Biodentine, Journal of Endodontics. (2018) 44, no. 7, 1186–1190, 10.1016/j.joen.2018.04.009. 29861064

[12] Prado M. , Santos Júnior H. M. , and Rezende C. M. , et al.Interactions Between Irrigants Commonly Used in Endodontic Practice: A Chemical Analysis, Journal of Endodontics. (2013) 39, no. 4, 505–510, 10.1016/j.joen.2012.11.050. 23522546

[13] González-López S. , Camejo-Aguilar D. , Sanchez-Sanchez P. , and Bolaños-Carmona V. , Effect of CHX on the Decalcifying Effect of 10% Citric Acid, 20% Citric Acid, or 17% EDTA, Journal of Endodontics. (2006) 32, no. 8, 781–784, 10.1016/j.joen.2006.02.006. 16861082

[14] Scelza M. Z. , Iorio N. L. P. P. , and Scelza P. , et al.Cytocompatibility and Antimicrobial Activity of a Novel Endodontic Irrigant Combining Citric Acid and Chlorhexidine, Journal of Dentistry. (2022) 125, 10.1016/j.jdent.2022.104278, 104278. 36058346

[15] Song M. , Park M.-G. , Kwak S.-W. , Kim R. H. , Ha J.-H. , and Kim H.-C. , Pilot Evaluation of Sealer-Based Root Canal Obturation Using Epoxy-Resin-Based and Calcium-Silicate-Based Sealers: A Randomized Clinical Trial, Materials. (2022) 15, no. 15, 10.3390/ma15155146, 5146. PMC9332451 35897577

[16] Srivastava A. , Yadav D. S. , Rao M. , Rao H. M. , Arun A. , and Siddique R. , Evaluation of Push-Out Bond Strength of BioRoot RCS and AH Plus After Using Different Irrigants: An In Vitro Study, Journal of Conservative Dentistry. (2020) 23, no. 1, 26–31, 10.4103/JCD.JCD_223_20. PMC7657418 33223637

[17] Basrani B. , Endodontic Irrigation, Chemical Disinfection of the Root Canal System, 2015, Springer International Publishing AG.

[18] Gesi A. , Raffaelli O. , Goracci C. , Pashley D. H. , Tay F. R. , and Ferrari M. , Interfacial Strength of Resilon and Gutta-Percha to Intraradicular Dentin, Journal of Endodontics. (2005) 31, no. 11, 809–813, 10.1097/01.don.0000158230.15853.b7. 16249724

[19] Pane E. S. , Palamara J. E. A. , and Messer H. H. , Critical Evaluation of the Push-Out Test for Root Canal Filling Materials, Journal of Endodontics. (2013) 39, no. 5, 669–673, 10.1016/j.joen.2012.12.032. 23611388

[20] Ungor M. , Onay E. O. , and Orucoglu H. , Push-Out Bond Strengths: The Epiphany-Resilon Endodontic Obturation System Compared With Different Pairings of Epiphany, Resilon, AH Plus and Gutta-Percha, International Endodontic Journal. (2006) 39, no. 8, 643–647, 10.1111/j.1365-2591.2006.01132.x. 16872459

[21] Stiegemeier D. , Baumgartner J. C. , and Ferracane J. , Comparison of Push-Out Bond Strengths of Resilon With Three Different Sealers, Journal of Endodontics. (2010) 36, no. 2, 318–321, 10.1016/j.joen.2009.10.026. 20113800

[22] Retana-Lobo C. , Tanomaru-Filho M. , Guerreiro-Tanomaru J. M. , Benavides-García M. , Hernández-Meza E. , and Reyes-Carmona J. , Push-Out Bond Strength, Characterization, and Ion Release of Premixed and Powder-Liquid Bioceramic Sealers With or Without Gutta-Percha, Scanning. (2021) 2021, 10.1155/2021/6617930, 6617930. PMC8121597 34040690

[23] Donnermeyer D. , Vahdat-Pajouh N. , Schäfer E. , and Dammaschke T. , Influence of the Final Irrigation Solution on the Push-Out Bond Strength of Calcium Silicate-Based, Epoxy Resin-Based and Silicone-Based Endodontic Sealers, Odontology. (2019) 107, no. 2, 231–236, 10.1007/s10266-018-0392-z. 30276580

[24] Carrilho M. R. , Carvalho R. M. , and de Goes M. F. , et al.Chlorhexidine Preserves Dentin Bond *in vitro* , Journal of Dental Research. (2007) 86, no. 1, 90–94, 10.1177/154405910708600115. PMC2248723 17189470

[25] Ghatole K. , Indi S. , Diwanji P. , Janavathi , Hambire A. , and Thimwala A. , Comparative Evaluation of N-Acetylcysteine and Chlorhexidine as Final Irrigation on the Push-Out Bond Strength of Different Sealers: An In Vitro Study, Endodontology. (2022) 34, no. 3, 180–183, 10.4103/endo.endo_81_22.

[26] Gupta P. R. , Aggarwal S. D. , Kshirsagar S. P. , Bhargava K. , Rai V. , and Chawla M. , Comparative Evaluation of Push-Out Bond Strength of Resin-Based Sealer and Mineral Trioxide Aggregate-Based Sealer After Using Normal Saline and 2% Chlorhexidine as a Final Irrigant: An In Vitro Study, Endodontology. (2016) 28, no. 1, 32–37, 10.4103/0970-7212.184337.

[27] Vianna M. E. , Gomes B. P. F. A. , Berber V. B. , Zaia A. A. , Ferraz C. C. R. , and de Souza-Filho F. J. , In Vitro Evaluation of the Antimicrobial Activity of Chlorhexidine and Sodium Hypochlorite, Oral Surgery, Oral Medicine, Oral Pathology, Oral Radiology, and Endodontology. (2004) 97, no. 1, 79–84, 10.1016/S1079-2104(03)00360-3. 14716261

[28] Prado M. , Gusman H. , Gomes B. P. F. A. , and Simão R. A. , Scanning Electron Microscopic Investigation of the Effectiveness of Phosphoric Acid in Smear Layer Removal When Compared With EDTA and Citric Acid, Journal of Endodontics. (2011) 37, no. 2, 255–258, 10.1016/j.joen.2010.11.011. 21238813

[29] Shekhar S. , Mallya P. L. , Ballal V. , and Shenoy R. , To Evaluate and Compare the Effect of 17% EDTA, 10% Citric Acid, 7% Maleic Acid on the Dentinal Tubule Penetration Depth of Bio Ceramic Root Canal Sealer Using Confocal Laser Scanning Microscopy: An In Vitro Study, F1000Research. (2022) 11, 10.12688/f1000research.127091.1, 1561. PMC9978241 36875990

[30] Turk T. , Kaval M. E. , and Şen B. H. , Evaluation of the Smear Layer Removal and Erosive Capacity of EDTA, Boric Acid, Citric Acid and Desy Clean Solutions: An In Vitro Study, BMC Oral Health. (2015) 15, no. 1, 10.1186/s12903-015-0090-y, 104. PMC4558635 26335205

[31] Khalil I. , Naaman A. , and Camilleri J. , Properties of Tricalcium Silicate Sealers, Journal of Endodontics. (2016) 42, no. 10, 1529–1535, 10.1016/j.joen.2016.06.002. 27523906

[32] Olivieri J. G. , García Font M. , Stöber E. , de Ribot J. , Mercadé M. , and Duran-Sindreu F. , Effect of Manual Dynamic Activation With Citric Acid Solutions in Smear Layer Removal: A Scanning Electron Microscopic Evaluation, Journal of Dental Sciences. (2016) 11, no. 4, 360–364, 10.1016/j.jds.2016.01.006. PMC6395180 30894998

[33] Kokkas A. B. , Boutsioukis A. C. , Vassiliadis L. P. , and Stavrianos C. K. , The Influence of the Smear Layer on Dentinal Tubule Penetration Depth by Three Different Root Canal Sealers: An In Vitro Study, Journal of Endodontics. (2004) 30, no. 2, 100–102, 10.1097/00004770-200402000-00009. 14977306

[34] Xu H. , Ye Z. , Zhang A. , Lin F. , Fu J. , and Fok A. S. L. , Effects of Concentration of Sodium Hypochlorite as an Endodontic Irrigant on the Mechanical and Structural Properties of Root Dentine: A Laboratory Study, International Endodontic Journal. (2022) 55, no. 10, 1091–1102, 10.1111/iej.13800. PMC9545283 35833329

[35] Dewi A. , Upara C. , Chaiariyakul D. , and Louwakul P. , Smear Layer Removal From Root Canal Dentin and Antimicrobial Effect of Citric Acid-Modified Chlorhexidine, European Endodontic Journal. (2020) 5, no. 3, 257–263, 10.14744/eej.2020.38258. PMC7881384 33353912

[36] Leelapornpisid W. , Dewi A. , Chaiariyakul D. , Upara C. , and Louwakul P. , Antibiofilm Effect of Citric Acid-Modified Chlorhexidine Gluconate on a Dual-Species Biofilm, Chiang Mai Dental Journal. (2021) 42, no. 3, 42–48.

[37] Razmi H. , Bolhari B. , Dashti N. K. , and Fazlyab M. , The Effect of Canal Dryness on Bond Strength of Bioceramic and Epoxy-Resin Sealers After Irrigation With Sodium Hypochlorite or Chlorhexidine, Iran Endodontic Journal. (2016) 11, no. 2, 129–133. 10.7508/iej.2016.02.011 PMC4841349 27141222

[38] Neelakantan P. , Sharma S. , Shemesh H. , and Wesselink P. R. , Influence of Irrigation Sequence on the Adhesion of Root Canal Sealers to Dentin: A Fourier Transform Infrared Spectroscopy and Push-Out Bond Strength Analysis, Journal of Endodontics. (2015) 41, no. 7, 1108–1111, 10.1016/j.joen.2015.02.001. 26008114

